# Therapeutic approaches to colorectal cancer *via* strategies based on modulation of gut microbiota

**DOI:** 10.3389/fmicb.2022.945533

**Published:** 2022-08-04

**Authors:** Maohua Chen, Wei Lin, Nan Li, Qian Wang, Shaomi Zhu, Anqi Zeng, Linjiang Song

**Affiliations:** ^1^School of Medical and Life Sciences, Chengdu University of Traditional Chinese Medicine, Chengdu, China; ^2^School of Medical Technology and Engineering, Fujian Medical University, Fuzhou, China; ^3^Institute of Translational Pharmacology and Clinical Application, Sichuan Academy of Chinese Medical Sciences, Chengdu, China

**Keywords:** gut microbiota, colorectal cancer, diet, probiotic, prebiotic

## Abstract

Colorectal cancer (CRC) ranks third in terms of global incidence and second in terms of death toll among malignant tumors. Gut microbiota are involved in the formation, development, and responses to different treatments of CRC. Under normal physiological conditions, intestinal microorganisms protect the intestinal mucosa, resist pathogen invasion, and regulate the proliferation of intestinal mucosal cells via a barrier effect and inhibition of DNA damage. The composition of gut microbiota and the influences of diet, drugs, and gender on the composition of the intestinal flora are important factors in the early detection of CRC and prediction of the results of CRC treatment. Regulation of gut microbiota is one of the most promising new strategies for CRC treatment, and it is essential to clarify the effect of gut microbiota on CRC and its possible mechanisms to facilitate the prevention and treatment of CRC. This review discusses the role of gut microbiota in the pathogenesis of CRC, the potential of gut microbiota as biomarkers for CRC, and therapeutic approaches to CRC based on the regulation of gut microbiota. It might provide new ideas for the use of gut microbiota in the prevention and treatment of CRC in the near future and thus reduce the incidence of CRC.

## Introduction

Colorectal cancer (CRC) is a malignant tumor occurring in the colon or rectum in the lower gastrointestinal tract. According to data from a 2020 survey by the World Health Organization, CRC ranks third and second in terms of incidence and mortality among all cancers and third and second in terms of incidence among men and women, respectively (Sung et al., 2021). The latest data show that the number of new cases of CRC in the world is as high as 193 million in 2020, and it is expected that the number of new cases of CRC in the world will increase to 2.5 million by 2035, surpassing common cancers such as liver cancer and gastric cancer (Sung et al., 2021).

The evolution of CRC is a complex multistep process. With the continuous developments and changes in people’s lifestyles toward Western lifestyles, the incidence and mortality rates of CRC are rising (Chen W. et al., 2016), which seriously threatens human health. The pathogenesis of CRC is complex and is due to synergistic effects of multiple factors. Its risk of occurrence is not only associated with mutations in multiple genes but also depends on the immune status of the patient, which is positively correlated with the intestinal flora. In recent years, with the intensification of research on intestinal microorganisms, research on the use of immunotherapy for treating CRC has been widely carried out in clinical settings. Further research on gut microbiota is likely to provide a promising approach for improving the efficacy of existing chemotherapy drugs, reducing their toxicity, and improving the sensitivity of immunotherapy (Gopalakrishnan et al., 2018).

**FIGURE 1:**
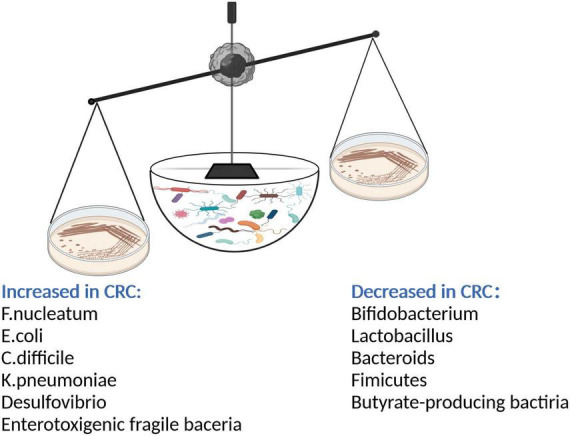
After gut microbiota imbalance, intestinal probiotics such as *Bifidobacterium*, *Lactobacillus*, Bacteroides, butyrate-producing bacteria decreased; the number of pathogenic bacteria such as Fusobacterium nucleatum, Enterotoxigenic fragile bacteria, *Escherichia coli*, *Clostridium difficile*, *Klebsiella pneumonia*, Desulphurization increased. The pathogenic bacteria secreted a large number of toxic factors to damage intestinal epithelial cells and promote the occurrence of CRC.

Gut microbiota promote intestinal repair by promoting cell proliferation and differentiation, which are essential for maintaining the integrity of the intestinal barrier (Krishnan et al., 2015). There is growing evidence that the large and complex gut microbiota interact via a complex mechanism with substances in the surrounding microenvironment (Geller et al., 2017). Gut microbiota play a critical role not only in maintaining the homeostasis of the intestinal environment but also in the maturation and sustainable development of the immune system (Thaiss et al., 2016). Various factors such as age, diet, lifestyle, and genotype act synergistically to cause changes in the structure and abundance of intestinal microbial populations (Seesaha et al., 2020). Imbalances in gut microbiota and disorders of flora population structure such as reductions in populations of beneficial bacteria and the presence of *Clostridium*, *Enterobacter*, and other pathogenic bacteria result in microecological imbalances and thereby disrupt cell DNA, activate carcinogenic signaling pathways, and ultimately affect the development of CRC (Brennan and Garrett, 2016; Figure 1).

In recent years, fecal microbiota transplantation has improved the benefits of treatment for patients with many diseases (Lu et al., 2019). Fecal microbiota transplantation involves the transfer of fecal microbiota from a healthy donor to the patient’s intestine to correct bacterial imbalances and restore intestinal homeostasis. By reconstructing the intestinal flora, it has an antitumor immune effect. The mechanisms by which the intestinal flora affects carcinogenesis, inflammation, and immune and therapeutic responses at the local level have been revealed in existing studies (Kim et al., 2020; Wang and Li, 2022). However, the specific mechanisms whereby microorganisms at the distal intestinal epithelial barrier regulate the occurrence of CRC, immunity, and physiological functions of multiple organs are not clear.

Although methods for “accurately” regulating intestinal microflora are still in their infancy, there is a lack of ideal screening methods, and there have been few studies on treatment techniques using intestinal microflora, there is no doubt that the regulation of intestinal microflora will be more widely used for overall health improvements and adjuvant therapy (Liu et al., 2018). Therefore, in the era of precision medicine, it is still necessary to promote research on techniques for the steady-state reconstruction of intestinal microflora to further clarify the relationship between intestinal microflora and CRC. Personalized treatment tailored according to the genetic background of the patient and the individual intestinal microflora promises to reduce the risk of CRC and improve patient prognosis, which will have a wide range of positive effects on public health.

## Interactions between intestinal flora and colorectal cancer

There are abundant microbial communities in the human body, which are mainly concentrated in the nose, mouth, skin, and intestines, especially the intestines. The total number of intestinal microorganisms may reach 100 trillion. The intestinal flora is an increasingly important factor in human digestion, metabolism, immunity, and other processes (Valdes et al., 2018), and it is therefore referred to as another “organ” of the human body. The intestinal flora can regulate immune function, synthesize vitamins, maintain the intestinal barrier, and promote the metabolism and absorption of materials. The pathogenesis of CRC is a multistep and multifactor process (Dahmus et al., 2018). It is therefore necessary to elucidate the mutual effects of the intestinal flora and gastrointestinal tract and their roles in the pathogenesis of CRC and to provide potential therapeutic targets and new ideas for the prevention and treatment of CRC.

**FIGURE 2:**
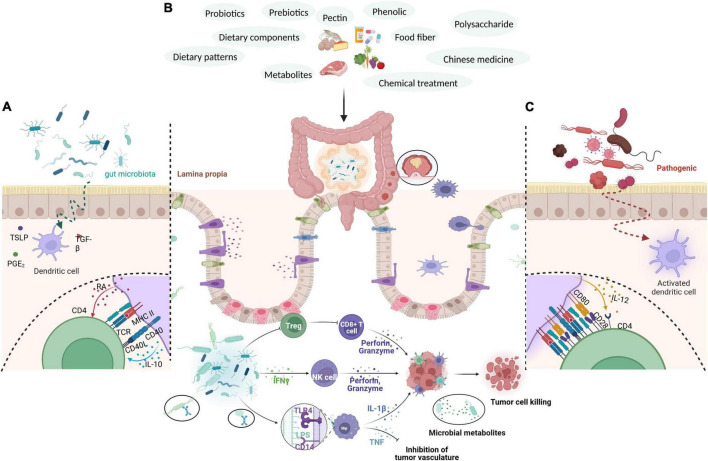
Through external intervention measures, such as the use of probiotics, prebiotics, polysaccharides, pectin, polyphenols, plant fibers, bacterial metabolites, drugs, and diet, the richness of intestinal flora is increased, intestinal immunity is regulated, the immune response of the body is enhanced, the secretion of anti-cancer cytokines is increased, and the colorectal mucosa is protected, thereby effectively intervening in CRC. **(A)** Gut microbiota produce substances such as PGE2, TGF-β, and TSLP to inhibit the maturation of dendritic cells (DCs). Immature DCs give weak co-stimulatory signals and secrete cytokines such as RA and IL-10 to induce CD4T cells to differentiate into Treg cells. **(B)** Gut microbiota induces the formation of T cells, secretes INF-γ, and activates NK cells. After contacting the intestinal epithelium, it enhances the phagocytic activity of macrophages through the receptor pathway, promotes cytokines such as TNF and IL, stimulates the body’s immune response, and exerts an anti-tumor effect. **(C)** Intestinal pathogenic bacteria penetrate the intestinal epithelium to activate DCs. The activated DCs express strong costimulatory ligands and induce CD4T cells to differentiate into effective TH1 and TH17 cells.

The intestinal flora is divided into probiotics, conditionally pathogenic bacteria, and pathogenic bacteria. Probiotics, such as *Bifidobacterium* and *Lactobacillus*, widely exist in common fermented milk and have various beneficial effects on health (Sokol et al., 2020). Probiotics are mostly specific anaerobic bacteria and are dominant in the intestinal flora. Conditional pathogens are mainly facultative anaerobic bacteria, such as *Enterobacter* and *Enterococcus*, whereas pathogenic bacteria include *Salmonella*, *Proteus*, and pathogenic *Escherichia coli*. The dominant intestinal flora at the phylum level mainly comprises Firmicutes, Bacteroidetes, and Actinobacteria (Chen J. et al., 2022; Mohammadi et al., 2022). The normal intestinal flora plays a significant role in the homeostasis of the intestinal environment, including participating in the protection, structure formation, and metabolism of the intestinal epithelium. Imbalances in the intestinal flora can change the intestinal microenvironment, including changes in intestinal epithelial genes, the occurrence of inflammatory reactions, the production of toxic metabolites, and damage to the intestinal epithelial barrier (Gagniere et al., 2016). All these changes are potential pathogenic factors with regard to CRC (Dinan and Cryan, 2017). For example, the intestinal flora plays a crucial role in the development of CRC by inducing abnormal immune responses in colon tissue to weaken the intestinal epithelial barrier and form a specific immune microenvironment (Si et al., 2021; Figure 2).

Ecological imbalances in the intestine promote the development of CRC, and the microbial community at a lesion site is significantly different from that in the adjacent healthy mucosa. The toxic substances produced by bacterial metabolism during an imbalance in the intestinal flora mainly comprise hydrogen sulfide, reactive oxygen species, active nitrogen species, and bile acid metabolites, which can damage intestinal epithelial cells, reduce the protective barrier effect of intestinal epithelial cells, and induce cell proliferation. Carcinogenic metabolites can also induce DNA damage in intestinal epithelial cells and thus produce mutations and induce the development of CRC (Avuthu and Guda, 2022). Studies have shown that fecal microbiota transplantation can induce polyp formation and change the local immune environment in mice (Sokol et al., 2020). Researchers have attempted to use probiotics to prevent and treat CRC. Different bacterial species play different roles in stimulating inflammation, inducing the release of proinflammatory toxins, increasing the production of reactive oxygen species, changing signaling pathways, and preventing immune function against CRC (Pennisi, 2013). According to research, probiotics, prebiotics, and synbiotics have been developed (Amitay et al., 2020). In addition to the effect of probiotics on treatment response, clinical trials (Gagliani et al., 2014; Panebianco et al., 2018) have examined the effects of probiotics on treatment-related toxicity. Studies have shown that in colon cancer patients the administration of 5-fluorouracil (5-FU) in combination with *Lactobacillus rhamnosus* can help improve the oral mucosa and alleviate diarrhea (Mego et al., 2013). In a mouse model of colon cancer induced by inflammation, supplementation with probiotics can reduce tumor cell proliferation and the number of tumors, downregulate the activation of nuclear factor (NF)-κB, and promote the activity of probiotics in the intestine (Osterlund et al., 2007). Recent studies in mice with colon cancer have shown that oral intake of probiotic supplements containing *Lactobacillus helveticus* can reduce the number of interleukin (IL)-17^+^ T cells and inhibit proliferation and tumor formation, which may be achieved via a change in the intestinal microflora (Osterlund et al., 2007).

In recent years, intestinal microorganisms have been proved to interact with the central nervous system indirectly via the gut–brain axis and thus affect the chemistry and functioning of the central nervous system. The gut–brain axis is a two-way communication system that integrates nerve, hormone, and immune signals between the central nervous system and intestinal tract (Dinan and Cryan, 2017). The intestinal tract can synthesize and secrete various neuroactive substances that can penetrate the blood–brain barrier and affect the brain (Di et al., 2019). Similarly, neuroactive molecules derived from the brain can affect the intestinal tract via the sympathetic and parasympathetic systems or humoral pathways (Petra et al., 2015); examples include substance P, calcitonin gene-related peptide, and neuropeptide Y (Evans et al., 2013). Microorganisms can affect the body’s stress level and behavior by participating in two-way regulatory pathways within the gut-brain axis, including neural signaling pathways, immune responses, the intestinal mucosal barrier, and 5-hydroxytryptamine and tryptophan metabolic pathways, and play a role in tumor growth (Miller et al., 2008; Holzer and Farzi, 2014; Alpert et al., 2021). Bacteria in the intestinal microenvironment can regulate cancer. Probiotics with metabolic activity can change the chemical structures of chemotherapy drugs by increasing or decreasing their activity and thereby changing their effective local concentration. It is increasingly clear that synbiotic microorganisms promote overall immunity (Rong et al., 2019). In addition, evidence suggests that these microorganisms may increase susceptibility to certain cancers by direct effects due to their local existence in the tumor microenvironment or systemic effects via distant microbial communities such as those in the gut and skin (Popa et al., 2022). The latter effects are particularly related to the ability of intestinal microbial regulation to respond to the toxicity of traditional chemotherapy drugs and immunotherapy and may affect the prognosis of patients (Vieira et al., 2013).

Drug therapies for CRC are mainly divided into basic chemotherapy drugs, targeted drugs, biological agents, and traditional Chinese medicinal prescriptions (Zhang et al., 2020). In recent years, numerous studies have been devoted to investigating the intestinal flora and its metabolites associated with the incidence, development, and treatment of CRC in order to better utilize the intestinal flora to prevent and treat CRC. Biological agents, targeted therapies, and traditional Chinese medicinal prescriptions when combined with chemotherapy can achieve ideal curative effects in clinical treatment and provide various treatment methods for CRC patients. Therapeutic strategies targeting the intestinal flora have shown great potential (Sivan et al., 2015). The modulatory role of the intestinal flora in the treatment of CRC includes enhancing the sensitivity of patients to immunotherapy, reducing adverse reactions to chemotherapy drugs, and reducing radiation damage. However, it is worth drawing attention to the fact that the onset of CRC is occult, and public awareness of its symptoms is low. Many patients are already in the late stage of disease when diagnosed, which results in poor therapeutic effects and a poor prognosis. Therefore, screening for CRC should also receive attention.

## Regulatory effect of probiotics on intestinal flora in colorectal cancer

The intestinal flora is a complex microbial community symbiotic with the human body and has attracted the attention of an increasing number of researchers over recent years. In this way, the study of probiotics has become more in-depth (Sobhani et al., 2011; Zhu et al., 2014). In recent years, the regulatory role of probiotics in the immune system and their antitumor characteristics have gradually become clear. Probiotics are live microorganisms that are beneficial to the body, of which the most widely used are *Lactobacillus*, *Bifidobacterium*, and *Clostridium butyricum* (Zhong et al., 2014). Bacterial strains may be responsible for detecting and degrading potential carcinogens and producing short-chain fatty acids (SCFAs) that affect cell death and proliferation (Eslami et al., 2019). Probiotic strains reduce the production of anti-inflammatory cytokines or inhibit their expression and thereby prevent the occurrence of CRC (Silveira et al., 2020). Probiotics can additionally activate phagocytosis to eliminate early cancer cells (Gorska et al., 2019). In a comparison of postoperative complications in CRC patients, Bajramagic et al. (2019) discovered that complications occurred more often in patients who did not receive probiotic therapy, especially in cases of intestinal obstruction, which suggests that supplementation with probiotic therapy in patients given surgical treatment for CRC is beneficial. Probiotics are involved in the immune process by stimulating antibody responses and inhibiting monocyte proliferation. In addition, probiotics can increase levels of anti-inflammatory cytokines, such as IL-10 and IL-12, and decrease levels of proinflammatory cytokines, such as IL-1β and IL-6, which have strong anti-inflammatory activities (Gorska et al., 2019). It has been discovered that microecological agents (Bultman, 2016), especially probiotics, can exert antitumor effects by stimulating immune responses, enhancing the phagocytic activity of macrophages, and promoting the activity of cytokines such as TNF-α, INF-γ, IL-12, and NO.

The presence of *Lactobacillus* in the gut has been shown to play a role in the regression of cancer as a result of its effects on immune regulation, which can be used as evidence of interactions between bacterial metabolites, immune cells, and epithelial cells (Huang R. et al., 2022). *Lactobacillus* can not only downregulate the Wnt/β-catenin pathway (Ghanavati et al., 2020), but also upregulate TNF-related apoptosis-inducing ligand, downregulate the transcription expression of cyclin D1 and BIRC5a, reduce the proliferation of CRC cells, and induce apoptosis of CRC cells (Tiptiri-Kourpeti et al., 2016). Shadnoush et al. (2013) demonstrated that the stability of the intestinal environment can be maintained by probiotics. Probiotics are involved in the prevention of CRC by inhibiting allergies, controlling serum cholesterol levels, modulating immune function, and inhibiting the growth of potentially harmful bacteria. *In vitro* studies (Zhu et al., 2011) have also found that probiotics can affect malignant biological activities of CRC cells such as proliferation, apoptosis, and adhesion. For example, *Lactobacillus*-derived polyphosphate induces apoptosis in CRC cells; *Bacillus polyfermenticus* exhibits adhesion to the surface of colon adenocarcinoma cells and suppresses the proliferation of CRC cells in a dose-dependent manner; and *Bifidobacterium bifidum* inhibits the proliferation of the CRC cell lines HT-29, SW480, and Caco-2 and thereby alters cell morphology and suppresses tumor growth. *Lactobacillus acidophilus* inhibits pathogenic bacteria and maintains the balance of the intestinal flora. Other probiotics such as *Saccharomyces cerevisiae* and *Lactobacillus casei* can induce apoptosis by blocking the cell cycle, and *Bacillus polyfermenticus* and *Lactobacillus rhamnosus* are associated with regulation of the cell cycle (Lin et al., 2009) and inhibition of the progression of CRC.

De Moreno De Leblanc and Perdigon (2010) demonstrated that fermented milk weakened intestinal inflammatory responses by increasing the number of IL-10-secreting cells, promoting apoptosis, and reducing the activity of procarcinogenic enzymes. These effects put gut microbiota into an ideal state and reduced the risk of cancer. Wang T. et al. (2022) confirmed that increasing the expression of the tight junction proteins claudin-1, zonula occludens-1, and occludin and increasing the loss of goblet cells can prevent damage to intestinal barrier function. In addition, these changes not only inhibit the expression of proinflammatory cytokines, such as IL-1β, IL-6, IL-17A, IFN-γ, and TNF-α, and chemokines, such as C-X-C motif chemokine ligand-2, C-X-C motif chemokine ligand-3, C-X-C motif chemokine ligand-5, and chemokine ligand-7, but also increase the abundance of probiotics and decrease the abundance of pathogenic bacteria and thus reduce the extent of intestinal inflammation. Colitis caused by CRC can be alleviated via the abovementioned three methods.

Xu et al. (2021) found in a study of *Lactobacillus rhamnosus* Probio-m9 that it can inhibit the expression of p-signal transducer and activator of transcription-3 and p-Akt, improve the balance of the intestinal flora, especially *Akkermansia*, *Bifidobacterium*, and *Blautia*, and suppress effects leading to CRC-related carcinogenesis. In particular, the study indicated that *L. rhamnosus* Probio-m9 can affect the metabolism of microbiota, especially glucose metabolism, and pointed out that glucose metabolism plays a far-reaching role as a driving force in the occurrence of tumors. Chung et al. (2021) developed a drug delivery system using the lactic acid bacterium *Pediococcus pentosaceus*, which carried a promoter that enabled the accurate expression of a fused protein containing P8, which is a small protein obtained from *L. rhamnosus* CBT LR5. This system significantly reduced the volume of tumors and inhibited the growth of CRC.

*Bifidobacterium bifidum* inhibits the development and progression of CRC in multiple ways, including antibacterial activity, improving vitamin metabolism, and enhancing immunity (Frick and Autenrieth, 2013), and also enhances the efficacy of antitumor immunotherapy by improving the local microenvironment (Sivan et al., 2015). *Bifidobacterium* can promote the expression of the proapoptotic gene *BAX* in CRC, reduce the expression of Bcl-2, and promote apoptosis of cancer cells. The phospholipid wall on the intestinal surface, as a ligand of toll-like receptor, can also induce apoptosis of cancer cells and participate in regulation of tumor growth (Yamamoto and Matsumoto, 2016; Ghanavati et al., 2020). *Bifidobacterium* spp. are among the most commonly used probiotics and have favorable effects on various diseases. However, Yoon and Michels (2021) showed in a study that only a specific *Bifidobacterium* strain improved the effect of cancer treatment. *Bifidobacterium breve* JCM92 enhanced antitumor immunity by increasing the number of CD8^+^ T cells and CD8^+^ effector T cells and increasing the ratios of CD8^+^ T cells to regulatory T cells and CD8^+^ effector T cells to regulatory T cells. *B. breve* JCM92 enhanced the efficacy of antitumor treatment, including oxaliplatin and programmed cell death protein-1 (PD-1) blockade, via lymphocyte-mediated immune responses.

*In vitro* research also discovered (Arimochi et al., 2009) that *Salmonella typhimurium* can suppress the proliferation of SW480 human CRC cells and induce cell cycle arrest and apoptosis in CRC cells. A growing number of studies have proved that microbe-based therapy is an available means of regulating gut microbiota and treating CRC. However, according to current relevant research, the safety of this therapy is still the greatest challenge. A recent study (Zheng et al., 2020) demonstrated that a modified form of the prebiotic dextran that encapsulated spores of *Clostridium butyricum* could be specifically enriched in tumor tissues and increase the populations of anticancer SCFA-producing bacteria, such as *Eubacterium* and *Roseburia*, which not only increased the overall abundance of intestinal microbiota but also transformed tumor-promoting intestinal microbiota into antitumor microbiota. Moreover, the easily modified prebiotic also represented a therapeutic target for the combination of capecitabine and diclofenac. Most importantly, this study indicated that *C. butyricum* spores encapsulated by the prebiotic dextran have no obvious side effects and provide a highly reliable and effective strategy for the treatment of CRC and other gastrointestinal diseases (Liu et al., 2020).

**FIGURE 3:**
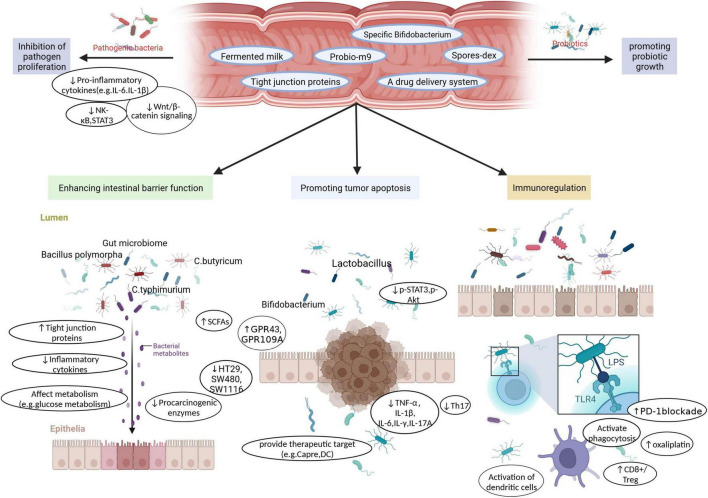
Probiotics as functional factors, such as Fermented milk, Probio-m9, specific Bifidobacterium, Spores-dex, Tight junction proteins, a drug delivery system, not only increase the proliferation of beneficial bacteria. It also inhibits the growth of pathogenic bacteria by inhibiting Pro-inflammatory cytokines, down-regulating Wnt/β-catenin signaling, inhibiting NK-κB and STAT3. Furthermore, various probiotic strains play a role in enhancing intestinal barrier function, promoting tumor cell apoptosis, and enhancing immune regulation through unique or common functions, to achieve the purpose of prevention or treatment of CRC.

Chen D. et al. (2020) pointed out that *C*. *butyricum* downregulated the Wnt/β-catenin signaling pathway, reduced levels of pathogens, such as *Clostridium* XIVb and *Clostridium* XI, and increased the abundance of *Lactobacillus*. Importantly, this study found that treatment with *C*. *butyricum* can change the metabolites of microorganisms, such as secondary bile acids and SCFAs, and activate the G protein-coupled receptors GPR43 and GPR109A to inhibit the development and progression of CRC (Figure 3).

## Regulatory effect of prebiotics on intestinal flora in colorectal cancer

In recent years, the use of prebiotics as an adjuvant treatment strategy for CRC patients has been gradually promoted. In 1995, Gibson and Roberfroid defined a prebiotic as an undigested or indigestible food component that selectively stimulates the proliferation and metabolism of a range of bacteria and thereby benefits the health of the host. The activated bacteria should be naturally beneficial, such as bifidobacteria and lactobacilli. In 2008, the Food and Agriculture Organization of the United Nations added to the definition of prebiotics at a special conference on prebiotics: prebiotics are inanimate food components that promote the growth of beneficial intestinal bacteria while inhibiting harmful bacteria (Pineiro et al., 2008). In this section, we discuss the effects of prebiotics, including polysaccharides, pectin, phenolics, and food fiber, on CRC and the associated role of the intestinal flora (Table 1).

**TABLE 1:** This chart summarizes the effects of prebiotic on gut microbiota, and directly or indirectly plays a role in CRC.

Functional component	Effects on gut microbiota	References
Prebiotic	Prebiotics produce large amounts of SCFA through beneficial bacteria fermentation to improve the intestinal mucosal microenvironment.	Gagliani et al., 2014; Fruge et al., 2021
Polysaccharides	Polysaccharides can induce CRC cell apoptosis and inhibit CRC cell invasion, adhesion, and metastasis. It can also improve host flora and enhance host immunity.	Gagniere et al., 2016; Geller et al., 2017; Gopalakrishnan et al., 2018; Gorska et al., 2019; Ghanavati et al., 2020; Hazafa et al., 2020; Geng et al., 2021; Genua et al., 2021; Guo et al., 2021
Pectin	Pectin can improve the therapeutic effect of anti- CRC chemotherapy. It can also regulate intestinal flora and increase the abundance of butyric acid-producing bacteria.	Holzer and Farzi, 2014; Higashimura et al., 2016; Henriksen et al., 2017
Phenolic	Polyphenols can overcome the damage of CRC treatment drugs to normal cells and play a key role in the regulation of intestinal microorganisms.	Huang J. R. et al., 2019; Huang X. M. et al., 2019; Ji et al., 2020; Illescas et al., 2021; Huang R. et al., 2022
Food fiber	Gut microbiota ferments plant fibers to produce butyric acid, which can improve the therapeutic effect of drugs. Second, cellulose can also change the composition of flora.	Ji et al., 2019; Jiang et al., 2020; Jin et al., 2021; Jones and Molloy, 2021

Prebiotics can enable the production of a large amount of SCFAs via the fermentation of beneficial bacteria. SCFAs can not only provide 60–70% of the energy supply of mucosal cells and promote their growth but also acidify the gut environment, facilitate the production of mucin, improve the microenvironment of the intestinal mucosa, and reduce the migration of spoilage bacteria such as *Bacillus* spp., *Clostridium* spp., and *Escherichia coli.* (Eslami et al., 2019). SCFAs can also stimulate the expression of anti-inflammatory factors in dendritic cells and macrophages and induce the production of regulatory T cells and IL-10 (Wlodarczyk et al., 2021). In addition, prebiotics inhibit the release of TNF-α from inflammatory cells and exert anti-inflammatory effects (Genua et al., 2021). Moreover, SCFA analogs suppress the cycling of SW1116 cells in CRC, induce apoptosis, and reduce the expression of proto-oncogenes and cell proliferation, in which butyric acid is the main compound that plays a role (Rad et al., 2021).

### Polysaccharides

Polysaccharides are natural polymers that are composed of more than 10 monosaccharide units linked by glycosidic bonds and are widely obtained from animals, plants, algae, and microorganisms (Song et al., 2021). Polysaccharides act against tumor cells directly or indirectly. Their direct antitumor effects are reflected in their ability to induce apoptosis in cancer cells, block the cell cycle, and inhibit tumor cell invasion, adhesion, metastasis, and growth (Shi et al., 2013; Shang et al., 2022). Secondly, the indirect antitumor effects of polysaccharides involve enhancing immune function (Feng et al., 2019).

Polysaccharides play an important role in human immune regulation. Firstly, polysaccharides can increase the number of macrophages and thus improve the body’s immunity (Li et al., 2020b). Secondly, polysaccharides affect the proliferation ability, subgroup structure, and cytokine secretion activity of lymphocytes (Ji et al., 2020). Thirdly, polysaccharides can activate natural killer cells and enhance their cytocidal activity (Ao and Kim, 2020). Fourthly, polysaccharides can activate the human complement system (Tao et al., 2017). Polysaccharides can also prevent CRC and reduce its recurrence rate. With regard to the regulation of the intestinal flora by polysaccharides, clinical experiments have shown that polysaccharides can stimulate the activity of intestinal probiotics, inhibit harmful intestinal bacteria, improve the intestinal inflammatory environment, reduce the release of carcinogens, and affect signaling pathways involved in tumor formation, which thereby inhibits the occurrence of intestinal tumors (Yang W. et al., 2022). In addition, SCFAs, which are metabolites of polysaccharides fermented by the intestinal flora, can inhibit the proliferation of tumor cells, induce the synthesis of mucin, and strengthen tight junctions between epithelial cells, which thereby prevents inflammation and ultimately inhibits the growth of CRC (Tao et al., 2017; Xue et al., 2020).

Owing to the lack of carbohydrate-activated enzymes in the human body, most polysaccharides cannot be directly digested and absorbed by the human body (Yu Y. et al., 2018). However, carbohydrate-activated enzymes encoded by human intestinal microflora can convert oligosaccharides and polysaccharides into monosaccharides to produce easily absorbed SCFAs and other metabolites (Ping et al., 2020; Li et al., 2022). Therefore, the intestinal flora serves as a bridge between the host and polysaccharides. For example, Yu Z. et al. (2018) confirmed that an extracellular polysaccharide from *Rhizopus nigricans* can resist hydrolysis in gastric conditions and successfully reach the intestine. Probiotics in the intestine can selectively use this polysaccharide, and its metabolites can increase both the villus length and villus length/crypt depth ratio in colon tissue and the number of goblet cells capable of secreting acidic mucus and thereby enhance the functioning of the immune system. Pool-Zobel and Sauer (2007) confirmed that inulin-type fructans produce SCFAs via microbial fermentation in the human intestine, of which butyric acid and propionic acid can inhibit the growth of CRC cells and histone deacetylase. Moreover, butyric acid can effectively suppress the apoptosis of tumor cells, reduce the metastasis of cancer cells, and inhibit carcinogens by increasing the expression of enzymes involved in detoxification. Guo et al. (2021) confirmed that polysaccharides from *Ganoderma lucidum* could inhibit toll-like receptor-4/MyD88/NF-κB signal transduction, increase the number of goblet cells, the secretion of mucin 2, and the expression of tight junction proteins, inhibit macrophage infiltration, downregulate the expression of IL-1β, inducible nitric oxide synthase, and cyclooxygenase-2, and suppress lipopolysaccharide (LPS)-induced inflammatory signatures and the activation of mitogen-activated protein kinase (MAPK) in RAW264.7 macrophages, HT-29 intestinal cells, and NCM460 cells. Ji et al. (2019) confirmed that polysaccharides from *Ziziphus jujuba* cv. Muzao can not only improve the intestinal flora of the host but also maintain metabolic function. Li et al. (2020b) confirmed that an apple polysaccharide inhibited the nuclear aggregation of β-catenin and thereby inhibited the activation of the Wnt pathway in colon tissue. The abovementioned studies have proved that the administration of polysaccharides is a valid way to treat CRC. Rivera-Huerta et al. (2017) discovered that fructans from chicory and Mexican agave can decrease the concentration of TNF-α and prevent the formation of intestinal polyps, villous atrophy, and lymphatic hyperplasia, which may help to reduce the risk of CRC. Wang et al. (2018) found that carboxymethylated pachyman has strong antitumor activity. A treatment regimen comprising carboxymethylated pachyman in combination with 5-FU markedly increased the levels of probiotics, such as *Lactobacillus*, *Bacteroides*, butyrate-producing bacteria, and acetic acid-producing bacteria. It played a crucial role in regulating the ecological balance of intestinal microbiota. This research also showed that carboxymethylated pachyman can alleviate 5-FU-induced colon injury, and the mechanism may be related to regulation of the NF-κB, Nrf2/antioxidant responsive element, and MAPK/p38 pathways. Yuan et al. (2021) found that polysaccharides from *Albuca bracteata* also have strong antitumor activity. Experimental data show that *A. bracteata* polysaccharides either alone or in combination with 5-FU can reduce the concentration of cancer cells and the expression of β-catenin and related proteins. Moreover, treatment with *A. bracteata* polysaccharides can specifically enrich *Ruminococcus*, *Anaerostipes*, and *Oscillospira*, effectively improve the composition of gut microbiota, and improve the levels of fecal SCFAs, such as propionic acid, acetic acid, and butyric acid, to improve the antitumor efficacy of 5-FU and reduce adverse reactions to 5-FU. LPS is a significant product of intestinal gram-negative flora. In blood and cancer tissues from patients with CRC, the level of LPS increased significantly. This suggests that LPS is an important target for the treatment of CRC. Song et al. (2018) are developing a nanoparticle-based LPS-trapping system for promoting PD-1-based immunity in CRC. The development of this LPS-trapping system offers new ideas for the future treatment of the intestinal microbiome in CRC. Scientists can cultivate genetically engineered bacteria, which can secrete LPS traps, and polymyxin B can even be produced directly by *Bacillus polymyxa*. Because these bacteria can reasonably enter the host gut and then remove LPS and regulate the CRC microenvironment, they may achieve therapeutic effects.

### Pectin

Pectin (PC) is a kind of water-soluble food fiber that can protect the intestinal wall, activate useful bacteria in the intestine, adjust gastrointestinal function, remove intestinal waste, and prevent CRC. Palko-Labuz et al. (2021) found that novel enzymatically extracted PC from apples is an effective inhibitor of bacterial β-glucuronidase that can improve the cytotoxicity of irinotecan and promote the apoptosis of tumor cells. Their study suggested that PC can be used as an adjuvant for irinotecan treatment in patients with CRC and can reduce the side effects of chemotherapy and improve its therapeutic effect. Pi et al. (2020) confirmed that the PD-1/programmed death ligand-1 signaling pathway can modulate the metabolic activities of gut microbiota and thereby promote the immune monitoring function of the intestinal flora with regard to tumors. Another study (Zhang et al., 2021) showed that PC in combination with an anti-PD-1 monoclonal antibody can safely improve the efficacy of the anti-PD-1 antibody. Moreover, the use of PC can beneficially regulate the intestinal flora. PC increases the abundance of butyric acid and promotes the infiltration of T lymphocytes and thus enhances the efficacy of anti-PD-1 monoclonal antibodies.

### Phenolics

Polyphenol (Zhao and Jiang, 2021) is the general name for a group of chemical substances in plants, which are so named because they contain multiple phenolic groups. In the plant world, there are more than 6500 polyphenols or phenolic compounds and their derivatives, which generally exist in grains, vegetables, fruits, and dual-purpose plants used for both medicine and food (Huang X. M. et al., 2019). Polyphenols are the antioxidant substances that people ingest in the largest amounts from food every day. In humans and other animals, phenolic acids formed by the metabolism of polyphenols by the intestinal flora have biological activities such as scavenging free radicals, antioxidant and antitumor activities, bacteriostasis, and improving immunity (Yang Q. et al., 2022). Polyphenols can avoid damage caused by CRC drugs to normal cells by only acting on tumor cells, leaving normal cells unaffected. Therefore, anti-CRC immunotherapy based on polyphenols and intestinal microbiota (Zhao and Jiang, 2021) is worth investigating.

Luo et al. (2021) confirmed that ferulic acid was transformed into 4-vinylguaiacol in the intestine by gut microbiota. The activity of the latter was mediated by cell cycle arrest at the G1 phase and induction of apoptosis. Dobani et al. (2021) have shown that raspberry polyphenols can reduce DNA damage and protect the colonic epithelium by upregulation of the cytoprotective Nrf2/antioxidant responsive element pathway. Wang et al. (2020) pointed out that tea polyphenols (TPs) can exert chemopreventive and therapeutic effects on CRC via multiple pathways mediated by gut microbiota. For example, TPs can inhibit the proliferation of tumor cells via molecular pathways such as the phosphoinositide 3-kinase/Akt, MAPK, Wnt/β-catenin, and Janus kinase/signal transducer and activator of transcription pathways. TP is the general term for a class of polyhydroxyphenol compounds present in tea, of which the main members are catechin compounds (Wang X. et al., 2022). TPs may inhibit CRC metastasis by inhibiting matrix metalloproteinase-9, which is closely associated with tumor metastasis and plays an important role in the infiltration of tumor cells (Mileo et al., 2019). Augusti et al. (2021) presented the chemopreventive effect of jaboticaba peel powder in terms of reducing inflammation and CRC cell proliferation in a complex 3D model of CRC for the first time. Jaboticaba peel powder contains phytochemicals extracted from jaboticaba fruit peel, which are fermented by flora in the intestine to form phenolic compounds.

### Food fiber

Short-chain fatty acids, such as butyric acid and propionic acid, are produced via the fermentation of dietary fiber by the intestinal flora. A high concentration of butyric acid can inhibit the proliferation of CRC cells independently of the Warburg effect and inactivate a large number of carcinogenic signals by inhibiting histone deacetylases. In addition, butyric acid can promote the proliferation of regulatory T cells and strengthen intestinal immunity (Tuncil et al., 2020; Liu et al., 2022). It has been pointed out that fermentation of dietary fiber by the intestinal flora can produce butyric acid, which can improve the anti-tumor cell proliferation effect of drugs such as irinotecan (Encarnacao et al., 2018). Windey et al. (2015) confirmed that dietary addition of wheat bran extract, which is a prebiotic supplement containing arabinoxylan oligosaccharides, decreased colonic protein fermentation, changed the metabolic mode in the colon, and selectively promoted the growth of bifidobacteria. Moreover, it was found that cellulose changed the composition of the gut flora, increased the richness of probiotics, decreased the abundance of inflammatory intestinal bacteria, and inhibited colonic inflammation and tumor formation induced by treatment with azoxymethane/dextran sulfate sodium (Li Q. et al., 2021). Yoon and Michels (2021) found by a feasibility study that combined supplementation with fiber and calcium can prevent CRC via changes in the composition of intestinal microbiota. This is mainly because the consumption of inulin can increase the absorption of calcium.

## Regulatory effect of metabolites of intestinal microorganisms in colorectal cancer

As we have already mentioned with regard to the relationship between probiotics and CRC, *Clostridium butyricum* is a probiotic that produces butyric acid, which is a classical SCFA with strong anticancer activity. So how does butyric acid work? Data from Li et al. (2017) demonstrated that histone deacetylase relied upon the activity of butyric acid in the inhibition of the motility of CRC cells via deactivation of Akt/extracellular-regulated kinase signaling. Besides, Geng et al. (2021) discovered that butyric acid, in addition to acting as a histone deacetylase inhibitor and activating the G protein-coupled receptor GPR109a, not only significantly inhibited glucose transport and glycolysis in CRC cells by reducing the contents of glucose transporter 1 in the cell membrane and glucose-6-phosphate dehydrogenase in the cytoplasm but also dramatically improved the chemotherapeutic effect of 5-FU on tumor cells. It has been shown that blueberry and coffee bean extracts rich in hydroxycinnamic acids (HCAs) inhibit the growth of CRC-associated pathogenic bacteria, such as *Fusobacterium nucleatum*, *Bacteroides fragilis*, and *Prevotella intermedia* (Budryn et al., 2015; Zheng et al., 2019). In addition, HCAs can promote potential anti-oncogenes in *Bifidobacterium*, *Lactobacillus*, and *Roseburia* and reduce the occurrence of CRC (Leonard et al., 2021). Experiments by Li Z. et al. (2021) demonstrated that hydroxycarboxylic acid receptor-2 mediated tumor development and progression by promoting gut mucosal immunity and reducing the expression of oncogenes. However, HCAs are a double-edged sword because they not only promote the growth of *Bacteroides* spp. but also lead to organism-wide inflammatory reactions and metabolic disturbances, which induce the occurrence of CRC (Coman and Vodnar, 2020). Therefore, we need more research to confirm the relationship between HCAs and CRC.

Furthermore, it has been pointed out that trimethylamine *N*-oxide is a metabolite produced from choline and carnitine in food by intestinal microorganisms and may be an independent risk factor. Consequently, suppression of the formation of trimethylamine *N*-oxide is a potential drug target for reducing the risk of development of CRC (Oellgaard et al., 2017). Tryptophan (Ala, 2021) metabolites affect CRC via the indole pathway in intestinal microbiota. The same study also found that tryptophan metabolites can play a role in suppressing inflammatory enteritis and CRC via the 5-hydroxytryptamine system in intestinal pigment cells and the kynurenine pathway in immune cells and the intestinal lining (Table 2).

**TABLE 2:** The chart summarizes how metabolites of intestinal microorganisms affect CRC.

Metabolites of intestinal microorganisms	Effect	References
Butyrate	HDAC relied activity of butyrate on inhibition of CRC cells motility through deactivation of Akt/ERK signaling.	Kim et al., 2020
	Butyrate inhibits glucose transport and glycolysis in CRC cells by reducing the abundance of membrane GLUT1 and cytoplasm G6PD and improves the chemotherapy effect of 5 - FU.	Krishnan et al., 2015
Hydroxycinnamic acid	HCA inhibit the growth of CRC-associated pathogenic bacteria, such as *Fusobacterium nucleatum*, *B. fragilis*, and *Prevotella intermedia.*	Li et al., 2018; Leonard et al., 2021
	HCA can promote the potential anti-oncogenes in *Bifidobacterium*, *Lactobacillus*, and *Roseburia*, and depress the occurrence of CRC.	Li Q. et al., 2021
	HCA receptor 2 mediates tumor development and progression by promoting gut-mucosal immunity and reducing oncogenes.	Li et al., 2017
Trimethylamine *N*-oxide Tryptophan	Inhibition of TMAO is a potential drug target for reducing the risk of CRC development	Li et al., 2022
	Tryptophan metabolites exert anti-CRC effects through the indole pathway, the serotonin system in the enterochromaffin cells, kynurenine pathway in the immune cells and internal lining.	Li et al., 2020a

## Regulatory effect of traditional Chinese medicine on intestinal flora in colorectal cancer

### Chinese medicine regulates the structure of the intestinal microflora and modulates the alteration of intestinal flora

#### Increasing the level of probiotics in the gut

After gavage with curcumin in rats, Weng and Goel (2020) discovered a significant improvement in the balance of the intestinal flora by quantifying fecal bacteria, with a decrease in levels of *Escherichia coli* and an increase in those of probiotics, such as *Bifidobacterium* and *Lactobacillus*. Besides, curcumin can upregulate the expression of peroxisome proliferator-activated receptor-γ and inhibit cancer cell proliferation (Zhang et al., 2016). Li et al. (2018) isolated three inulin-type fructans with high polymerization degrees from the roots of *Codonopsis pilosula* and found that they could stimulate an increase in bifidobacteria levels in the gut. Jin et al. (2021) demonstrated that *p*-cymene could stimulate the growth of probiotic bacteria such as isobacteria, bifidobacteria, and *Clostridium* IV in the intestinal tract.

#### Inhibiting the growth of pathogenic bacteria

Sui et al. (2020) found that YYFZBJS inhibited the production of regulatory T cells by altering intestinal bacterial populations, including those of *Bacteroides fragilis* and Lachnospiraceae spp. Although YYFZBJS itself had no suppressive effect on tumor cell proliferation, YYFZBJS-mediated alterations in regulatory T cells limited their growth, while β-catenin phosphorylation levels were reduced. Moreover, the researchers suggested YYFZBJS as a new potential drug option for the treatment of CRC. Shao et al. (2021) discovered that Xiao-Chai-Hu-Tang reversed dysregulation of gut microbiota by downregulating toll-like receptor-4/MyD88/NF-κB signaling and, in particular, by reducing the levels of *Parabacteroides*, *Blautia*, and Ruminococcaceae bacteria. The essential role of the intestinal flora in the treatment of CRC with depressive symptoms was elucidated.

Research has proved that berberine can reduce the population of *Clostridium nucleatum*, as well as inhibiting the overgrowth of *Pseudomonas aeruginosa* and *Staphylococcus* spp. caused by *C. nucleatum* colonization, which leads to a decrease in the secretion of immune factors and thus inhibits the occurrence of CRC (Yu et al., 2015; Chen H. et al., 2020). Furthermore, berberine can downregulate the expression of cyclooxygenase-2 and prostaglandin E2 and inhibit the invasion and metastasis of tumor cells (Liu et al., 2015). Ji et al. (2019) showed that polysaccharides from *Ziziphus jujuba* cv. Muzao reduced the growth of pathogenic bacteria such as *Sclerotinia sclerotiorum*, alleviated dysbiosis of the intestinal flora caused by colitis, and thus prevented the onset of tumors. *Paris polyphylla* and its active components polyphyllin VI and pennogenin 3-*O*-β-chacotrioside can inhibit the growth of *Fusobacterium* and thus inhibit the migration and invasion of CRC cells. Moreover, polyphyllin VI and pennogenin 3-*O*-β-chacotrioside can have a significant inhibitory effect on *Bifidobacterium*. For this reason, when they are used in the treatment of CRC, supplementation with bifidobacteria is required to overcome this inhibitory effect (Lin et al., 2021). Triterpenoid phytosaponins derived from gibberellins can reduce the amount of sulfate-reducing bacteria in the intestine of APCMin/ + model mice, strengthen the intestinal epithelial mucosal barrier, and simultaneously downregulate the expression of Src in the intestinal mucosa and other proteins and inhibit tumor growth (Chen L. et al., 2016).

### Chinese medicine increases the abundance of gut microflora and improves the intestinal microecological balance

Chen et al. (2018) researched the effect of *Panax notoginseng* saponins on the intestinal microflora in mice. The results indicated that these saponins markedly increased the abundance and diversity of the intestinal flora, especially *Akkermansia* spp., and that ginsenoside compound K, which is a metabolite of *P. notoginseng* saponins produced by gut microflora, had anti-CRC activity, suppressed the proliferation of tumor cells, and regulated the homeostasis of the intestinal flora. Terasaki et al. (2021) found that fucoxanthin remarkably reduced levels of Bacteroidales, Rikenellaceae, Lachnospiraceae, and Erysipelotrichaceae spp. in the intestine of mice and that the abundances of *Lactobacillus*, *Lactococcus*, *Bifidobacterium*, and several butyrate-producing bacteria increased. This was accompanied by a change in the ratio of Firmicutes to Bacteroidetes. These findings indicated that fucoxanthin induced changes in the bowel microbiota and the tumor microenvironment, which may be an important mechanism responsible for its potential anticancer effect. Zhu et al. (2021) found that alisol B 23-acetate decreased the levels of pathogenic bacteria, such as *Klebsiella*, *Citrobacter*, and *Akkermansia*, and increased the populations of beneficial bacteria, such as *Bacteroides*, *Lactobacillus*, and *Alloprevotella*. Wang et al. (2017) identified that the regulation of the microbial balance by American ginseng contributed to the maintenance of intestinal homeostasis, as evidenced by restoration in the form of population shifts and recovery from dysbiosis among gut microbial populations, with significant changes in abundance in the families Bacteroidaceae and Porphyromonadaceae.

Another study revealed that Sini Decoction (Wang et al., 2021) effectively increased the diversity of the intestinal flora, markedly increased the number of dominant intestinal bacterial groups, including *Bacillus coagulans*, *Lactobacillus*, *Xylophilus*, and *Bifidobacterium*, and reduced levels of pathogenic bacteria, including sulfate-reducing bacteria and *Bacteroides fragilis*, and thereby protected the colonic mucosal barrier and regulated the intestinal microecological balance. Quxie Capsule (Sun et al., 2020) notably reduced the proportion of harmful intestinal bacteria, such as *Shigella* and Cyanobacteria, and increased the abundance of beneficial bacteria, such as *Actinobacteria* and *Lachnospiraceae*. Wu Mei Wan (Jiang et al., 2020) dramatically increased the content of Firmicutes, decreased the content of Bacteroidetes, and downregulated the expression of p65, IL-6, and p-signal transducer and activator of transcription-3 in cancer cells in CAC mice.

### Chinese medicine participates in immune function by regulating intestinal microflora

*In vitro* experiments demonstrated that *Dendrobium* Sonia polysaccharide could activate the immune system by increasing the viability and phagocytic capacity of macrophages. These experiments indicated that it may be an effective macrophage activator that can promote immune function in mice with cyclophosphamide-induced immunosuppression by promoting the release of the cytokines IL-6, TNF-α, and interferon-γ. In addition, *Dendrobium* Sonia polysaccharide significantly alleviated dysregulation of the intestinal microbiota in mice with cyclophosphamide-induced immunosuppression and increased the proportion of probiotic bacteria (Liu et al., 2019; Xie et al., 2019). *Bifidobacterium* can play a protective role in TNF-α-induced inflammatory responses in Caco-2 cells via the NF-κB and p38/MAPK pathways (Nie et al., 2020). *Astragalus* can modulate the structure of the gut flora and regulate immunity by inhibiting activation of NF-κB, which in turn decreases high expression levels of proinflammatory genes and weakens intestinal inflammatory responses mediated by proinflammatory factors (Zhang et al., 2019). Baicalin is converted into baicalein by human intestinal microorganisms. On comparing the antiproliferative effects of baicalein and baicalin, experiments have confirmed that the latter has a more potent effect. Moreover, baicalin was able to reduce damage to the organism by activating the Wnt/β-catenin signaling pathway and promoting the expression of Bax protein, which leads to apoptosis of cancer cells (Wang et al., 2015, 2019).

**FIGURE 4:**
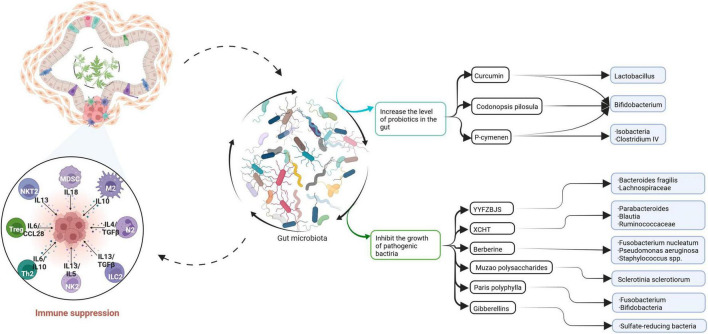
This paper summarizes the relationship between traditional Chinese medicine and gut microbiota and CRC. Curcumin, *Codonopsis pilosula*, and *P*-cymenen increased the abundance of intestinal probiotics. Some Chinese herbal extracts or prescriptions can inhibit the growth of pathogenic bacteria. Traditional Chinese medicine increases the richness of gut microbiota, thereby enhancing the body’s immunity and playing an anti-CRC role.

Sankaranarayanan et al. (2021) identified five bacterial species, namely, *Flavonifractor plautii*, *Bacillus glycinifermentans*, *Bacteroides eggerthii*, *Eubacterium eligens*, and *Olsenella scatoligenes*, which can degrade quercetin and produce different bioactive metabolites. Some of these species have been proved to have anti-CRC cell antiproliferative effects. In particular, *B*. *glycinifermentans* and *F*. *plautii* were prominent in terms of their ability to degrade quercetin, and the authors considered them to have excellent potential for the development of anti-CRC probiotics for the human gut. The combination of microencapsulated *Bifidobacterium bifidum* and *Lactobacillus gasseri* with quercetin, which was invented by Benito et al. (2021), suppressed the Wnt/β-catenin signaling pathway in the mouse colon and thereby inhibited the progression of CRC. Another study revealed that Gegen Qinlian decoction not only regulated components of the intestinal microbiota and promoted the antitumor activity of anti-PD-1 antibodies (Li et al., 2020a) but also enhanced the immunity of patients and protected the functioning of the intestinal barrier (Lv et al., 2019; Figure 4).

## Regulatory effect of chemical treatments on intestinal flora in colorectal cancer

Chemotherapeutic approaches for treating CRC have received increased attention in recent years. Many cytotoxic agents are regularly used in combination, for example, 5-FU in combination with L-oxaliplatin and capecitabine in combination with either oxaliplatin (XELOX regimen), irinotecan (XELIR regimen), cyclooxygenase-2 inhibitors, cetuximab, or bevacizumab. According to investigation data, however, the therapeutic efficacy of chemotherapy frequently leads to failure of CRC treatment as a result of drug resistance.

There is a growing amount of empirical data suggesting that the use of metformin increases the level of SCFA-producing intestinal microorganisms. Metformin (Jones and Molloy, 2021) indirectly affects SCFAs by altering the composition of the gut flora and has a role in the treatment of CRC. In their experimental study, (Huang J. R. et al., 2019) produced liposomal irinotecan, which remarkably reduced the level of prostaglandin E2 in colon tissue and significantly reduced the rate of bifidobacterial infection in comparison with irinotecan.

Aspirin-based gut microbe therapy has been investigated. In a randomized clinical study, aspirin was proved to increase the abundance of *Prevotella*, *Akkermansia*, and Ruminococcaceae spp. while reducing the abundance of *Bacteroides*, *Parabacteroides*, and *Dorea* spp., which is closely associated with the reduction of the risk of CRC achieved with aspirin (Prizment et al., 2020). A study by Diao and Cai (2020) indicated that intestinal microorganisms interacted with aspirin. On the one hand, gut microbes converted aspirin into inactive metabolites and enhanced its preventive effect against CRC. On the other hand, aspirin increased the abundance of some probiotic bacteria in the gut, such as bifidobacteria and lactobacilli. Another group of studies pointed out that biotransformation of aspirin by intestinal flora may be a source of 2,3-dihydroxybenzoic acid and 2,5-dihydroxybenzoic acid, which have an inhibitory effect on the growth of CRC cells and play critical roles in the anticancer effect of aspirin (Sankaranarayanan et al., 2020).

Other studies have shown that fecal microbiota transplantation can safely alleviate intestinal inflammation, maintain the integrity of the colonic epithelium, and reduce intestinal damage caused by FOLFOX therapy, which is a 5-FU-based chemotherapeutic regimen (Chang et al., 2020).

## Regulatory effect of diet on intestinal flora in colorectal cancer

### Different dietary components, microbiota, and colorectal cancer

#### Fat

Wan et al. (2019) organized low-fat, medium-fat, and high-fat diet intervention experiments on 217 healthy young adults and found that the diversity of intestinal microbiota in young adults on a high-fat diet was significantly reduced. The abundance of *Alipipes* and *Bacteroides* increased significantly, while that of *Faecalibacterium* was significantly reduced. Furthermore, it was found in related animal experiments that a high-fat diet promoted the secretion of bile acids in mice. An imbalance in the intestinal microenvironment and an increase in levels of secondary bile acids will lead to deterioration of the functioning of large intestinal epithelial cells and damage to the mucosal layer and thereby increase the risk of CRC in mice (Ridlon et al., 2016). Moreover, when mice were given a high-fat diet, the abundance of Lactobacillales decreased and that of the *Clostridium* subcluster XIVa increased (Higashimura et al., 2016). In addition, after rats were switched from a low-fat diet to a high-fat diet, the diversity of intestinal microbiota decreased markedly, and the relative abundance of Firmicutes increased greatly (Taira et al., 2015).

#### Protein

The influences of different sources of protein on the composition of intestinal microbiota and their roles in the occurrence of CRC are significantly different. Studies have indicated that the intake of chicken protein can increase the abundance of *Lactobacillus* in rat intestines, whereas soy protein can increase the abundance of *Ruminococcus* and *Bacteroides* and reduce the relative abundance of *Lactobacillus* (Zhu et al., 2017). The intake of fish meat can more effectively prevent the occurrence of CRC, whereas the intake of red meat will increase the risk of CRC (Farvid et al., 2021).

#### Carbohydrates

Fermentation of complex carbohydrates (e.g., dietary fiber) by intestinal microflora produces high levels of SCFAs, such as butyric acid. High levels of SCFAs may reduce the risk of the development of CRC. Butyric acid induces apoptosis in colon cells, inhibits the activity of CRC cells by blocking the expression of genes regulating histone deacetylase (Li et al., 2017), and also activates glycoisomerization via a cAMP-dependent mechanism.

#### Micronutrients

Recent work has demonstrated that micronutrients can influence the development and progression of CRC by modulating the structure of the intestinal microbial community.

Vitamin D (Tabatabaeizadeh et al., 2018) plays a significant role in maintaining the stability of gut microbiota and regulating intestinal probiotics and the intestinal barrier regulated by *Akkermansia muciniphila*. Vitamin D (Tabatabaeizadeh et al., 2018), as a cell differentiation inducer, affects telomerase activity and exerts an antitumor effect via its receptors on cells. An experimental study by Zhou et al. (2020) discovered that a sufficient amount of vitamin D can effectively reverse CRC. This is because vitamin D has a strong regulatory effect on the integrity of the intestinal barrier mediated by *A*. *muciniphila*. This finding may suggest a new target for the therapy of CRC via intestinal microbiota. Vitamin E (Yang et al., 2021) is a highly potent fat-soluble intracellular antioxidant that can inhibit the formation of free radicals and the activity of protein kinase, which is closely associated with the growth and differentiation of tumor cells. Vitamin E in the form of δ-tocotrienol and its metabolite 13’-carboxychromanol increased levels of potentially beneficial *Lactococcus* and *Bacteroides*. Yang et al. (2021) supported the hypothesis that this metabolite may play a role in δ-tocotrienol’s anticancer activity and modulation of gut microbes. A sufficient amount of vitamin A can increase the diversity of intestinal microbiota, and vitamin A plays an indirect role in regulating the intestinal barrier and immune responses. All-*trans*-retinoic acid (Cantorna et al., 2019) is the most active form of vitamin A and induces apoptosis of tumor cells and has a therapeutic effect in CRC. The intestinal flora acts as a mediating effector. Vitamin B_12_ (Lurz et al., 2020) causes changes in the abundance of gut microbiota, such as a decrease in *Lactobacillus* and an increase in *Bacteroides*.

In contrast, Phipps et al. (2020) discovered from the effect of iron on the gut flora in CRC that supplementation with oral iron preparations in iron-deficient CRC patients may lead to noteworthy changes in gut microbiota, the growth of harmful microorganisms, and exacerbation of the disease process. Hence, micronutrients themselves and their effects on the intestinal microecological balance may contribute to the development of CRC, and their route of intake and disruptive effect on the intestinal flora need to be taken into account when supplementing with micronutrient preparations, especially iron preparations.

### Different dietary patterns, gut microbiota, and colorectal cancer

#### Mediterranean diet

Studies (Illescas et al., 2021) have shown that people in southern European countries with the Mediterranean diet are much less likely to suffer from CRC than those in other European and American countries. The Mediterranean diet is a representative model of a healthy diet that has been gradually promoted in recent years. Its characteristic feature is to encourage people to consume more nutritious fresh fruits and vegetables, beans, nuts, and whole grains, with fish and olive oil as the main sources of fat, supplemented by small amounts of dairy products, red meat, and wine. The Mediterranean diet is enriched with omega-3 polyunsaturated fatty acids (e.g., docosahexaenoic acid and eicosapentaenoic acid) and phytochemicals (e.g., polyphenols and flavonoids) that inhibit the expression of colonic proinflammatory factors and improve and regulate the intestinal microenvironment and thus reduce the risk of CRC (Illescas et al., 2021; Yammine et al., 2021). Yammine et al. (2021) found that dietary plant polyphenols in the Mediterranean diet have a significant effect on the prevention of CRC. These dietary plant polyphenols include apigenin, curcumin, epigallocatechin gallate, quercetin, rutin, and resveratrol. Further understanding of the mechanisms of polyphenol–microbiota interactions may provide further insights into strategies for the prevention or treatment of CRC. Metabolites produced by gut microbiota are more pharmacologically active than these natural molecules.

#### Vegetarian diet

The strictest vegetarian diet is defined as a diet that completely excludes meat and foods of animal origin (Sakkas et al., 2020). Currently, a limited number of prospective studies have demonstrated the positive effect of a vegetarian diet on the prevention of CRC (Orlich et al., 2015; Henriksen et al., 2017). At present, there is no definite conclusion on the specific mechanism by which a vegetarian diet can reduce the risk of CRC. Nevertheless, it is certain that reducing the intake of red meat and animal protein, together with the high contents of dietary fiber, monounsaturated and polyunsaturated fats, antioxidant vitamins, and phytochemicals such as polyphenols and carotenoids in a vegetarian diet, can increase the microbial diversity in the intestine, maintain intestinal homeostasis, and alleviate inflammation and oxidative stress in the intestine and thus reduce the risk of CRC (Hazafa et al., 2020; Sakkas et al., 2020).

#### Reducing intake of inflammatory foods

The term “inflammatory foods” refers to processed meat, red meat, viscera, refined flour, and sugary drinks, which are the foods most closely associated with CRC-related inflammation. Therefore, reducing the intake of inflammatory foods plays a role in the prevention and treatment of CRC. These foods have the opposite effects to “anti-inflammatory foods,” such as green leafy vegetables, dark yellow vegetables, whole grains, coffee, and fruit juice. Research has shown that dark green leafy vegetables (Fruge et al., 2021), rice bran (So et al., 2021), an increased intake of dietary indoles (Wyatt and Greathouse, 2021), vitamin D, calcium, folic acid, fruits, and other vegetables (Song et al., 2015) play substantial roles in the prevention of CRC. How does the composition of the diet prevent CRC and reduce the risk of CRC? After researching and analyzing the relationships between dietary interventions and CRC in recent years, the authors found that the mediating effect of gut microbiota is an important factor in dietary interventions and the occurrence of CRC. However, more work is needed to determine the effects of diet on gut microbiota. In the future, research on the individual differences and diversity of gut microbiota may contribute to a deeper comprehension of the treatment of CRC and may lead to deeper insights into the field of medicine.

### Processed products, gut microbiota, and colorectal cancer

Fernandez et al. (2018) developed an anthocyanin-rich sausage that prevented CRC after processing. Anthocyanins were the main components responsible for its anticancer effect and have been described as essential modulators of intestinal microbiota. For example, supplementation with anthocyanins in animal models of CRC was able to reduce the abundance of pathogenic bacteria (e.g., *Enterococcus* spp. and *Desulfovibrio* spp.) and increase the abundance of probiotics (e.g., *Eubacterium rectale* and *Faecalibacterium prausnitzii*) (Chen et al., 2018). They can therefore be used as an alternative to dietary interventions in CRC, especially in people who consume excessive amounts of meat products. Besides, Zhuang et al. (2021) observed in their experiments that CD8^+^ T cells were enriched in the intestinal microenvironment of mice with CRC treated orally with jujube powder, which increased the diversity of the intestinal microflora and the abundance of bifidobacteria and thus contributed to the production of butyric acid, which could inhibit tumor growth, in cecal contents. Moreover, the use of jujube powder to enrich the intestinal flora can improve the efficacy of chemotherapy for CRC, such as cyclophosphamide. Furthermore, the consumption of First Leaf, which consists of blackcurrant extract powder, lactoferrin, and lutein, and Cassis Anthomix 30, which comprises blackcurrant extract powder, as a novel prebiotic preparation had an anticancer effect in humans by increasing the abundance of intestinal probiotics, namely, *Lactobacillus* and *Bifidobacterium*, which may have been achieved by markedly decreasing the fecal pH and the activity of bacterial β-glucuronidase (Molan et al., 2014). In addition, the beneficial effect of *Boswellia serrata* resin extract in alleviating microbial dysbiosis in azoxymethane/dextran sulfate sodium-induced CRC was demonstrated by alterations in the composition of gut microbiota in the form of an increase in the amount of *Clostridium perfringens* and a decrease in the abundance of Bacteroidetes (Chou et al., 2017; Table 3).

**TABLE 3:** The chart summarizes how different dietary components, dietary patterns, and some elaboration products affect gut microbiota and thus play a role in preventing CRC.

Diet	Nutrient or Food	Effect	References
Dietary components	Fat	High-fat diet reduces the diversity of intestinal microbiota.	Ridlon et al., 2016; Rivera-Huerta et al., 2017; Rong et al., 2019; Rad et al., 2021
	Protein	High protein diet increases the abundance of intestinal microbiota.	Sakkas et al., 2020; Sankaranarayanan et al., 2020
	Carbohydrates	Dietary fiber produces large amounts of SCFAs after fermentation in the intestine.	Kim et al., 2020
	Vitamin D	Regulation of *A. muciniphila*-mediated intestinal barrier integrity.	Seesaha et al., 2020; Sankaranarayanan et al., 2021
	Vitamin E	Suppressing the formation of free radicals and the active of protein kinase.	Shadnoush et al., 2013
	Vitamin A and Vitamin B12	Influencing the composition of the intestinal flora.	Shao et al., 2021; Shang et al., 2022
Dietary patterns	Mediterranean diet	ω-3 polyunsaturated fatty acids, phytochemicals inhibit the expression of colonic inflammatory factors.	Shi et al., 2013; Si et al., 2021
	Vegetarian diet	Reducing the intake of red meat and animal protein, adequate dietary fiber, etc., which can increase the microbial diversity of the intestine.	Smith et al., 2007; Sivan et al., 2015; Silveira et al., 2020; So et al., 2021
Elaboration products	Anthocyanin-rich sausage	It serves as a dietary intervention alternative to CRC, where anthocyanins can regulate intestinal flora.	Song et al., 2018
	Jujube powder	Not only does it increase the number of Bifidobacteria, but it also boosts the efficiency of CRC chemotherapy.	Sun et al., 2020
	Blackcurrant products	It not only reduces the fecal pH, but also reduces the activity of the bacterial β-glucuronidase.	Sung et al., 2021
	*Boswellia serrata* resin extract	It increases the proportion of *Clostridium perfringens* and decreases the ratio of Bacteroidetes.	Tabatabaeizadeh et al., 2018

## Conclusion

At present, the study of microbial communities has been applied in related fields of human disease and has achieved success. However, the role and significance of microbial communities in treatment have not been fully studied, and we still face many unresolved problems. Therefore, it is necessary to conduct further research on current topics to verify existing findings and develop treatment strategies. It is worth noting that current research on microbial regulation of anti-CRC therapy mostly focuses on mice, and how to apply the resulting academic findings in clinical practice remains a challenge. Although the pathological and immune responses of mice implanted with human microbiota are similar to those of humans, there are still differences (Smith et al., 2007).

In the future, if the most beneficial microbial compositions under various clinical conditions can be identified, it will be possible to use microbial compositions as biomarkers, diagnostic tools, or therapeutic targets and develop microbial therapies that can not only enhance the effects of anticancer therapies but also reduce their systemic toxicity. Therefore, treatment interventions targeting microbial groups will become one of the next areas of focus for precise and personalized treatment of cancer. This may lead to breakthroughs in various areas of medicine and not only support the development and production of immunotherapies or innovative vaccines for cancer treatment but also improve drug delivery in other intestinal diseases and prevent and reduce inflammation. A clinical trial of this strategy has the potential to improve the feasibility of using probiotics as comprehensive drug carriers in non-invasive cancer treatment in humans and as suitable adjuvants in traditional anticancer therapy.

## Author contributions

MC, LS, and SZ: conceptualization. MC, WL, and NL: writing—original draft preparation. MC, AZ, QW, SZ, and LS: writing—review and editing. AZ and LS: visualization. All authors have read and agreed to the published version of the manuscript.
